# Role of Mechanical Factors in the Morphology of the Primate Cerebral Cortex

**DOI:** 10.1371/journal.pcbi.0020022

**Published:** 2006-03-24

**Authors:** Claus C Hilgetag, Helen Barbas

**Affiliations:** 1 School of Engineering and Science, International University Bremen, Bremen, Germany; 2 Boston University, Boston, Massachusetts, United States of America; 3 Boston University School of Medicine, Boston, Massachusetts, United States of America; 4 New England Primate Research Center, Harvard Medical School, Southborough, Massachusetts, United States of America; UFR Biomédicale de l'Université René Descartes, France

## Abstract

The convoluted cortex of primates is instantly recognizable in its principal morphologic features, yet puzzling in its complex finer structure. Various hypotheses have been proposed about the mechanisms of its formation. Based on the analysis of databases of quantitative architectonic and connection data for primate prefrontal cortices, we offer support for the hypothesis that tension exerted by corticocortical connections is a significant factor in shaping the cerebral cortical landscape. Moreover, forces generated by cortical folding influence laminar morphology, and appear to have a previously unsuspected impact on cellular migration during cortical development. The evidence for a significant role of mechanical factors in cortical morphology opens the possibility of constructing computational models of cortical develoment based on physical principles. Such models are particularly relevant for understanding the relationship of cortical morphology to the connectivity of normal brains, and structurally altered brains in diseases of developmental origin, such as schizophrenia and autism.

## Synopsis

How are the characteristic folds of primate brains formed? New answers to this old question support the idea that folding occurs as nerve fibers connect the brain's different surface regions. The fibers pull together regions that are strongly connected, while unconnected regions drift apart. Furthermore, as the brain develops before birth and its surface expands, folding may affect the passage of new neurons into different regions, influencing the brain's architecture. These findings underscore the role of mechanical forces in shaping the normal brain. Moreover, the findings suggest that changes in brain shape in developmental diseases, such as schizophrenia and autism, may result from changes in the connections.

## Introduction

The popular image of the human brain is closely associated with the intricate folds of the cerebral cortex. This cortical landscape has been described, measured, and interpreted since the age of phrenology [1]. The mammalian cerebral cortex evolved and expanded tangentially [2–4], and folded to accommodate a large surface area, measuring 1,600–2,000 cm^2^ in humans, three times larger than the inner surface of the skull [1,5].

Classic studies described cortical morphology [6,7], frequently with reference to its geometric regularity [8–10]. Various hypotheses were proposed for the development of convolutions, such as active growth [7], pressure and friction of expanding cortex tangentially against the skull or underlying brain structures [9], and mechanical bulging from unequal regional expansion [6,11–15]. Other concepts suggested that cortical buckling results from differential laminar growth [16–18], or that convolutions are shaped through attached axonal fibers [19]. More specifically, it has been proposed, but not yet rigorously tested, that the 3-D shape of the brain reflects the viscoelastic tension exerted by axonal fibers [5]. According to this hypothesis, global competition of mechanical forces results in the formation of gyri between densely linked regions, and sulci between weakly connected or unconnected regions. The axonal tension hypothesis is particularly attractive, since it implies that the characteristic cortical morphology arises automatically from the interconnections of different cortical areas, without the need for individual specification of convolutions. Moreover, cortical folding through axonal tension implicitly achieves a desirable reduction in the volume of cortical fibers [5].

Mechanical factors have been invoked in most models for the development of cortical convolutions [5,6,9,11–19], but see also a discussion on active growth of convolutions [7]. Mechanical forces may also have a role in observed trends in laminar cortical morphology [5,10] and the deformation of neurons [10] and blood vessels [20] in different parts of the cortical landscape. Nevertheless, the rapid progress of research into the genetic control of brain development during recent decades has sidelined mechanical concepts in favor of genetic models for explaining the morphology of the convoluted cortex [21]. Beause of the apparent complexity of cortical morphology and the fact that many aspects of cortical development are still poorly understood, there is a great need for establishing systematic and reliable quantitative data on the architecture of the brain in order to evaluate different morphological concepts.

Here we address two questions related to the role of mechanical factors in cortical morphology: (1) Is the overall pattern of connections in the adult convoluted cortex consistent with the hypothesis that axonal tension underlies the formation of cortical convolutions? (2) Are systematic variations in adult cortical architecture related to cortical convolutions? Both questions are addressed through the analysis of quantitative data for connections and architecture of the prefrontal cortex in adult nonhuman primates. Quantitative tract tracing in animals with a convoluted cortex offers the most detailed and reliable information currently available about the density and trajectories of cortical projections. Retrograde tract tracing, in particular, is ideal for this purpose because each projection neuron is labeled by transport of a tracer through individual axons from the injection site back to the parent cell body. A count of labeled neurons, therefore, provides a good estimate of the number of axons that link a given pair of cortices. Over the last two decades we have systematically accumulated quantitative data on connections of the prefrontal cortex in a gyrencephalic nonhuman primate, the rhesus monkey (e.g., [22–24]). In addition, we have obtained quantitative architectonic data on all prefrontal areas in the same species [25]. Using these databases we provide quantitative evidence consistent with a key role of mechanical factors in three interrelated aspects of cortical morphology: (1) the formation of cortical convolutions through axonal tension; (2) the shaping of laminar morphology through cortical folding; and (3) a nonuniform distribution of neurons in cortical layers that may result from the interaction of tensile and compressive folding forces with neuronal migration during development. Some of these findings were previously reported in conference proceedings [26].

## Results

We considered the role of mechanical factors in three interrelated aspects of cortical morphology by investigating: (1) whether the overall pattern of connections in the adult convoluted cortex is consistent with the hypothesis that axonal tension contributes to the formation of cortical convolutions; (2) how cortical folding affects the morphology of layers in gyri and sulci; and (3) how cortical folding affects the thickness and neuronal content of cortical columns, as elaborated below. Because tension in axons is central to discussing the role of projections in the formation of cortical convolutions, we include background information on tension in cells, and the mechanics that explain the formation of gyri and sulci in the cortex as a result of axonal tension. These processes lead to a prediction for the layout of cortical projections in the adult brain.

### Axonal Tension and Cortical Folding

The cellular processes extending from neurons, axons and dendrites, exhibit a complex mixture of active and passive viscoelastic properties (reviewed in [5]). Importantly, neurites are capable of exerting small static forces for long periods due to their passive material properties [27]. Moreover, they can actively produce tension [27], and retract actively when released from external tension [28].

The axonal tension hypothesis posits that cortical convolutions result from axons pulling on neural tissue as they link areas. According to the hypothesis, gyri form between strongly connected areas as axons draw them together, and sulci arise between weakly linked or unconnected areas [5].

### Mechanical Model for the Formation of Convexities (Gyri) and Concavities (Sulci)

**Figure 1:**
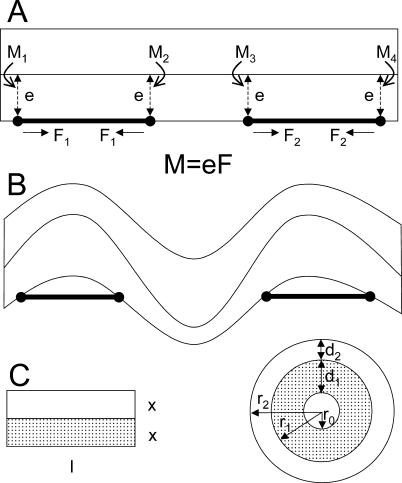
Mechanical Effects in Tissue Folding (A) A simple mechanical model illustrates the deformation of tissue subjected to tangential force (*F*_1_) at an eccentricity *(e)* from the points of attachment, causing a moment, *M.* This mechanical deformation is represented by the general relation *M* = e*F.* (B) The moment *M* causes the sheet to bend in a convex shape. The same principles apply to the second point of connection attachment, subjected to force *F*_2_, resulting in a convexity (gyrus) between connected regions, while a concavity (sulcus) forms between unconnected or weakly connected regions by default. (C) Relative thickness (i.e., the ratio of the thickness of upper to lower cortical layers), changes when a 2-D section of cortical tissue, on the left, is bent into an annulus, on the right. Specifically, the relative thickness of the bent upper layers, *d*_2_, is smaller than that of the lower layers, *d*_1_. A mathematical derivation of these relations is given in Materials and Methods (“Bending a slab of layered neural tissue”).

We present a simple mechanical model to illustrate the deformation of the cortical sheet subjected to tensile forces, as shown in Figure 1. A sheet of tissue subjected to a tangential force (*F*_1_) at a certain eccentricity *(e)* from the point of attachment causes a moment, *M* (Figure 1A), which can be calculated by the general relation *M* = e*F.* The moment *M* causes the sheet to bend in a convex shape. The same principle applies to the second point of connection attachment, subjected to force *F*_2_, resulting in a gyrus between connected regions, while a concavity (sulcus) forms between unconnected or weakly connected regions by default (Figure 1B).

This simple model explains the mechanics of the formation of convexities and concavities, and can be applied to any tissue that is bent, including cortical tissue. The model is independent of the exact size of the force, eccentricity, or moment generated, and so can be used to explain the formation of gyri and sulci without the need to specify the length of the axons, the amount of force exerted, or the eccentricity of the force, which are not known for the cortex. However, the specific parameters of length, force, etc., are important in modeling the characteristic shape of a given convolution, an issue that is beyond the scope of the present study.

### Prediction of the Axonal Tension Hypothesis

**Figure 2:**
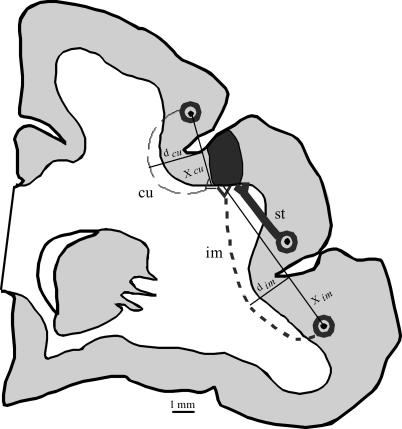
Three Types of Trajectories of Corticocortical Projections The diagram shows straight (st; thick solid line), intermediate (im; intermediate dashed line), and curved (cu; thin dashed line) projections that link a tracer injection site (dark spot) with different areas, shown in a coronal section through the rhesus monkey prefrontal cortex. The curvature *(c)* of the trajectories is determined by the relation *c* = *d*/*x,* in which *x* is the minimum distance (in 3-D Euclidean space) between projection origin and termination, and *d* the maximum deviation of the actual projection from the minimum distance path. These measures are indicated for examples of curved and intermediate projections, resulting in curvatures of *c_cu_* = 0.71 and *c_im_* = 0.26. Since the trajectory of the straight projection follows the minimum distance path, its curvature is zero. The thickness of the projections depicts the posited link between connection density and axonal trajectory. Axes of section: medial is to the left; dorsal, top.

According to the axonal tension hypothesis, the shape of the convoluted cortical landscape is produced by the global competition and resulting minimization of axonal tension. The process results in straightening of connections as convolutions form (compare Figure 2B and 2C in [5]). This aspect of the model makes a testable prediction for the end result of the folding process in the adult brain: most fiber trajectories in the adult cortex should be straight, particularly when dense. We tested this hypothesis by investigating the pattern of connections, as elaborated below.

### Relationship between densities and trajectories of projections

We tested the prediction that cortical folding involves a global straightening of fiber projections by systematically determining the trajectories of corticocortical projections from quantitative data of adult rhesus monkeys *(Macaca mulatta).* We focused specifically on projections that link prefrontal cortices of the gyrencephalic cortex of rhesus monkeys (map shown in Figure S1). The analyzed database included the densities of projections and the course of their axons obtained from retrograde tract-tracing experiments (23 injections in 21 animals). Injections of tracers were placed in half of all prefrontal areas and their subdivisions, and included lateral (dorsal and ventral area 46, dorsal area 8, and lateral area 12), orbitofrontal (orbital area 12, area 11, area 13, areas orbital periallocortex [OPAll]/orbital proisocortex [OPro]) and medial (area 32; medial area 9) prefrontal areas. The injection sites yielded a total of 289 projection sites with 123,866 projection neurons, which were found in all prefrontal cortices. For each injection case, data were normalized by expressing the number of labeled projection neurons in each projection site as a percentage of the total number of labeled neurons in all prefrontal cortices. This normalization provided information on the relative density of projections, which were identified by the architectonic areas in which the projection sites were localized. Projection trajectories were categorized into three classes based on the shortest possible path of axons between the projection site and the injection site in 3-D, as illustrated in a cross section through the prefrontal cortex in Figure 2. Projections were considered straight if they could follow the shortest possible path, intermediate if they were slightly deflected by an intruding sulcus, and curved if the projections were completely bent around a sulcus (see Figure 2 for details and quantitative classification of trajectories by curvature measure, *c;* see Materials and Methods).

#### Individual examples of connections.

**Figure 3:**
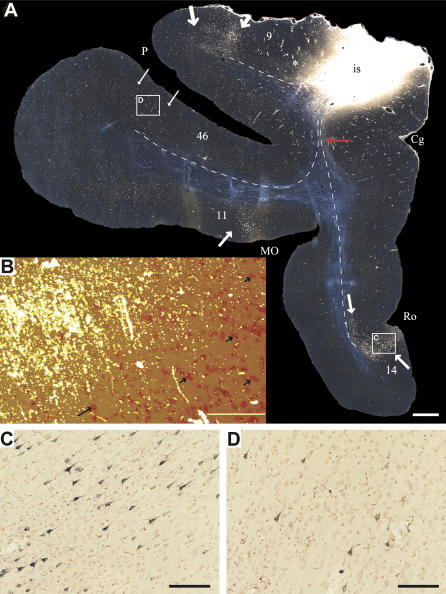
Example of Three Types of Trajectories Linking Prefrontal Cortices (A) Low-power darkfield photomicrograph of a coronal section through a middle level of the prefrontal cortex of a rhesus monkey brain showing an injection of HRP-WGA in area 9 (white area, “is”). Several projection sites can be seen (arrows). One projection with many labeled neurons is seen in the adjacent part of lateral area 9 (top, thick arrows); another projection is seen inferiorly in area 14 (medium arrows); a light projection is found in ventral area 46 below the principal sulcus (thin arrows), and another projection is seen in area 11. The dashed lines drawn in the white matter (appearing blue in darkfied) represent the shortest possible trajectory of axons in three of the projection sites. Axons linking lateral area 9 with the injection site (top) can take a fairly straight course. Labeled axons leaving the injection site are visible at this level (pink fibers in white matter), and some cross directly through layer VI (star). Axons linking area 14 with the injection site take a mildly deflected course through the white matter. Some labeled axons are seen exiting the injection site (pink fibers in white matter, below the depths of the cingulate [Cg] sulcus), and course inferiorly along the retrograde transport route to projection neurons. Axons linking ventral area 46 with the injection site travel below the principal sulcus (which is deep at this level) and must take a curved course. (B) High magnification of a site from an adjacent section to the one depicted in (A), showing that some axons (gold) coursing below the injection site take a straight course through the deep layers of the cingulate cortex. The approximate limit of the white matter is denoted by the large arrow (bottom left). The tissue was photographed under dark field illumination to show labeled axons in the white matter (gold, to the left of the large arrow), as well as axons coursing among neurons (red, some indicated by arrows), photographed under bright field illumination. The two photographs were merged. (C) Higher magnification of the mildly deflected projection in area 14 (from boxed area C in [A]) showing labeled neurons under brightfield illumination (blue pyramids). The labeled neurons at the top are in layer III, the ones at the bottom left corner are in layer V. (D) Higher magnification of the curved projection from ventral area 46 (boxed area D from [A]), shows a few labeled neurons in layer III (blue) under brightfield illumination. The section was counterstained with neutral red. Scale bars: (A) = 1 mm; (B–D), 100 μm. Orientation axes: medial, to the right; dorsal, on top. Cg, cingulate; MO, medial orbital; P, principal; Ro, rostral.

**Figure 4:**
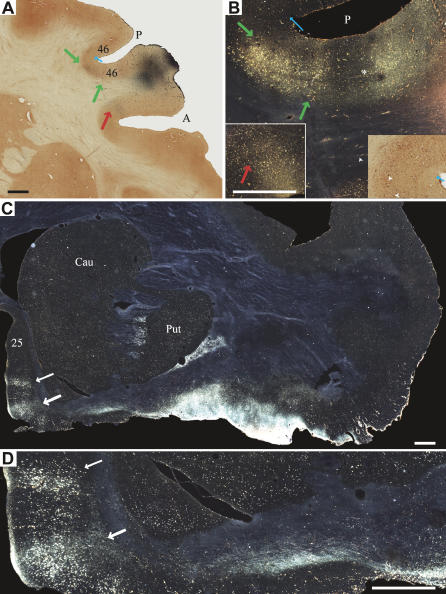
Examples of the Relationship of Projection Density to Axonal Trajectory (A) Low-power brightfield photomicrograph of a coronal section through the posterior prefrontal cortex of a rhesus monkey brain, showing the halo of an injection of HRP-WGA in ventral area 46, below the principal sulcus (dark area). Green arrows point to a projection site in area 46 in the depths of the lower bank of the principal sulcus (dark blotch). Another projection is found in the upper bank of the lower limb of the arcuate (“A”) sulcus (dark blotch, red arrow); axons from these projection sites can take a mildly deflected or straight course to the injection site. (B) Top: higher magnification under darkfield illumination of the depths and lower bank of the principal sulcus in the same section shows that the projection in ventral area 46 has many labeled neurons (gold, green arrows), and labeled axons (pink, white arrowhead) as they leave the halo of the injection site. Note that the dense projection abruptly terminates as the sulcus curves towards the upper bank of the principal sulcus, towards dorsal area 46. The magnified site at the lower right inset (blue arrow, brightfield illumination) shows only a few scattered labeled neurons in dorsal area 46 (white arrowheads), taken from the region with the corresponding blue arrows in (A, brightfield) and (B, darkfield). In contrast, the “straight” projection in the arcuate sulcus has many labeled neurons (lower left inset in [B], red arrow; darkfield illumination). The section was counterstained with neutral red. (C) Low-power darkfield photomicrograph of a coronal section through the caudal prefrontal cortex of a rhesus monkey brain showing the halo of the injection site in area OPAll/OPro (white area, bottom, center), and a resultant robust projection site of labeled neurons (white) arranged in a columnar pattern in area 25 (white arrows). The labeled fibers (white) around the putamen (Put) and in the internal capsule (interposed between the caudate and putamen, white) link the injection site with subcortical structures. (D) The columns of labeled neurons are shown at higher magnification. The inferiorly situated broad column of neurons (thick arrow) has a fairly straight course from the injection site, while the one above is deflected. Axons leaving the halo of the injection site are visible at this level (white fibers in white matter, which appears blue under darkfield illumination). Scale bars: (A), (C), 1 mm; (B), (D), 0.5 mm. Orientation axes: Medial, is to the left; dorsal, at the top.

Figures 3 and 4 show examples of the relationship of cortical convolutions to projections, and the shortest trajectory that axons can take to link pairs of areas. Figure 3A shows a cross-section through the prefrontal cortex, photographed under darkfield illumination, with an injection of the neural tracer horseradish peroxidase–wheat germ agglutinin (HRP-WGA) in area 9 (white area, “is”), and four projection sites with clusters of neurons (pink, arrows) labeled retrogradely from the injection site. The axonal trajectory to a site within area 14 inferiorly (arrow) is mildly deflected by the intruding cingulate sulcus (Cg), which is shallow at this point, and axonal curvature, *c,* within the section is about 0.05. More posteriorly, the cingulate sulcus is somewhat deeper and the axons become slightly more deflected, so the entire projection was classified as intermediate. A projection in the lateral part of area 9 is straight (thick arrow), as seen in the initial exit of axons from the injection site (pink fibers). Interestingly, some axons from the medial part of area 9 (injection site, white area) take a shortcut through the deep layers of the cortex (star), a pattern seen also in Figure 3B, where axons below the injection site cut across the deep layers of the cingulate cortex (at the level of the red arrow in Figure 3A). On the other hand, axons from ventral area 46 to the injection site must course below the principal sulcus, which is deep at this level, and thus must take a curved trajectory. The shortest possible trajectory of the axons of labeled neurons for each of three projection sites is depicted by a dashed line through the white matter (blue). As seen in Figure 3, there are more projection neurons in areas 9 and 14 (thick arrows) issuing straight and intermediate projections than in ventral area 46 (thin arrows), which is the origin of a curved projection. The differences in projection density are seen at higher magnification for area 14 (Figure 3C, blue labeled neurons) and ventral area 46 (Figure 3D), photographed under brightfield illumination from the corresponding boxed sites (in Figure 3A).

Figure 4A shows another example of a case with an injection of HRP-WGA in a lateral prefrontal area, ventral area 46 (black area shows the halo of the injection site). A dense cluster of projection neurons is found in ventral area 46 (green arrows, dark blotch), in the depths of the lower bank of the principal sulcus (P), and another moderately dense projection is found in ventral area 8, within the upper bank of the arcuate (A) sulcus (red arrow). In Figure 4B, the depths and lower bank of the principal sulcus were magnified and photographed under darkfield illumination. Many labeled neurons (gold) can be seen in ventral area 46 (Figure 4B, green arrows) and in the upper bank of the lower limb of the arcuate sulcus (Figure 4B, lower left inset, red arrow). The axonal trajectories for these projections are mildly deflected or straight, as assessed throughout their rostrocaudal extent (not shown). Label abruptly declines as the principal sulcus curves towards the upper bank (dorsal area 46), and only scattered projection neurons are found beyond the depths of the sulcus (Figure 4A and 4B, blue arrow; the magnified lower right inset shows the same site under brightfield illumination with only a few labeled neurons, white arrowheads). The weak projection from dorsal area 46 coincides with the curved trajectory that axons must take around the depths of the principal sulcus to the injection site in ventral area 46, whose core was anterior to this section (not shown).

Figure 4C shows another case with an injection of HRP-WGA in orbitofrontal area OPAll/OPro (white area shows label from the halo of the injection site). Axons emerging from the halo of the injection site take a mildly deflected course towards a projection site, composed of two columns of labeled neurons in area 25, shown also in Figure 4D at higher magnification. Axons linking the lower column (thick arrow) are only slightly deflected from a straight course, and axons linking the upper column are comparatively more deflected. The entire projection from area 25 in this case extended over several coronal sections, and was considered to be intermediate.

#### Cumulative data analysis.

**Figure 5:**
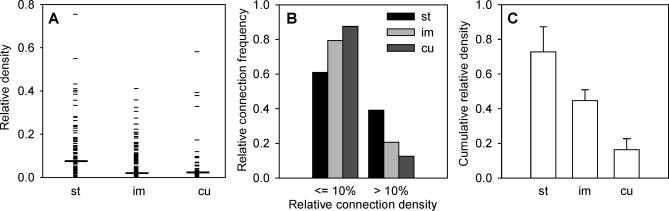
Relationship of Axonal Trajectories to Connections (A) Relative densities of individual projections in the different trajectory classes, straight (st), intermediate (im), and curved (cu). Horizontal lines denote median values. (Spearman's rank correlation of individual projections with trajectory class: ρ = −0.27, *p* < 0. 00001). (B) Relative distribution of projection trajectories. Relative density of projections (on the *x*-axis) was calculated as the number of corticocortical projection neurons in each area, divided by the total number of labeled neurons found for all areas in the prefrontal cortex for one injection case. The *y*-axis indicates the relative frequency of all projections in the given density interval (normalized by all projections in a trajectory class). The diagram demonstrates that weak projections (<10% of relative density) tended to form mostly curved, then intermediate and straight trajectories, whereas greater densities (>10%) resulted predominantly in straight trajectories. (C) Average cumulative density of straight, intermediate, and curved projections originating from individual prefrontal areas. The individual density values also followed the trend displayed here (rank correlation, ρ = −0.50, *p* < 0.0001).

We determined frequencies and central values of relative densities for all projections in prefrontal cortices, and categorized their trajectories as straight, intermediate, or curved (Figure 2) as described in Materials and Methods. We found that a large proportion of all projections (*n* = 289) were straight (*n* = 110) or only slightly deflected by intruding sulci (*n* = 131). Moreover, there was a significant decrease of individual projection densities from straight to intermediate to curved (rank correlation ρ = −0.27, *p* < 0.00001), with the median relative density of straight projections (0.075) outweighing the intermediate (0.020) or curved (0.023) by a factor of three (Figure 5A). We also computed the relative frequency of axonal trajectories across all cases, by summing all relative projection densities within the three trajectory classes and normalizing by the number of cases. The relative frequency was higher for straight trajectories, amounting to more than half of the total number of axons in all cases (53.7%), than for intermediate (32.8%) and curved (13.5%). Further, comparison of the distribution of projection densities showed that relatively sparse projections (of less than 10% relative density; Figure 5B) formed mostly curved projections, while stronger projections (>10% relative density) had predominantly straight trajectories (Figure 5B). We obtained very similar results when other statistical landmarks for separating sparse and dense projections were used, such as the average density of all projections (8%), or the median projection density (3.5%). In all cases, dense projections involved a greater number of straight than intermediate and a greater number of intermediate than curved trajectories.

In a global “tug of war” of tensional forces, individual projections may deviate from the expected relationship between projection density and trajectory. Indeed, a small number of projections with high relative densities followed curved or intermediate trajectories (Figure 5A). However, the summed density of all straight projections originating from a particular cortical area always outweighed the summed density of curved projections (Figure 5C). This is consistent with the idea that the global cortical landscape is shaped by the entire complement of connections.

#### Effect of Folding on Laminar Morphology

**Figure 6:**
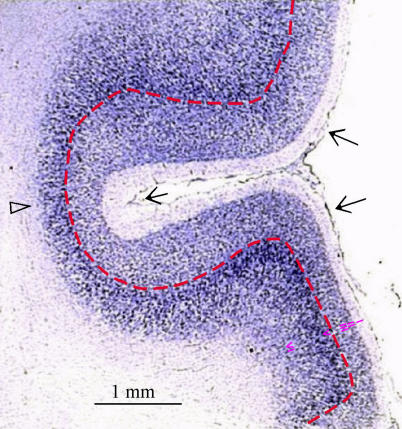
Effect of Cortical Folding on Absolute and Relative Thickness of Cortical Layers Coronal section through medial prefrontal cortex of a rhesus monkey brain in the anterior cingulate. Note the expanded deep layers (below dotted line demarcating the upper boundary of cortical layer V) in the gyral regions (large arrows), contrasted by reduced thickness of the deep layers at the bottom of the cingulate sulcus (arrowhead) and the expanded layer I (small arrow) in comparison with layer I in gyral cortex. The brain section was immunoreacted for NeuN, a neuronal marker (brown), and then stained for Nissl (blue).

Folding of the cortex produces tensile and compressive forces that affect cortical architecture, as expected for any material with a degree of flexibility and incompressibility (Figure 1). Folding results in systematic and significant biases in the morphology and relative thickness of cortical layers [5,10]. It follows from geometry that the thickness ratio of upper to deep cortical layers, that is, the relative laminar thickness, changes when a slab of cortical tissue is bent from straight into gyral or sulcal shape (Figure 1A–1C). For a formal mathematical expression of these relationships see Materials and Methods (“Bending a slab of layered neural tissue”); a more detailed physical exploration of these effects has also been provided [29]. In particular, in gyral regions, folding stretches the upper layers and compresses the deep layers tangentially, thereby reducing the relative thickness of the upper layers, and relatively expanding the deep layers in the radial direction. Opposite effects are expected for sulcal regions. These effects are readily apparent for sample sections throughout the cortex (for example, see Figure 6).

We obtained data on the absolute thickness of cortical layers for all prefrontal cortices using unbiased sampling procedures (for details see Materials and Methods) and calculated the relative thickness of each laminar compartment by normalizing its absolute thickness by total cortical thickness. We then tested quantitatively whether the predicted mechanical impact on laminar morphology can be identified as a global feature across all prefrontal cortices by examining all pairwise cross-correlations of the relative thickness of laminar compartments (I, II+III, IV, and V+VI) for 21 areas (see Materials and Methods). We found that the relative widths of layers I, II+III, and IV were all significantly and positively correlated, but significantly and negatively correlated with the thickness of layers V+VI (Table 1). This designated layers I, II+III, and IV as “upper” layers and V+VI as “deep” layers, and supported the prediction that cortical folding affects the upper and deep cortical layers in opposite directions.

**Table 1:**
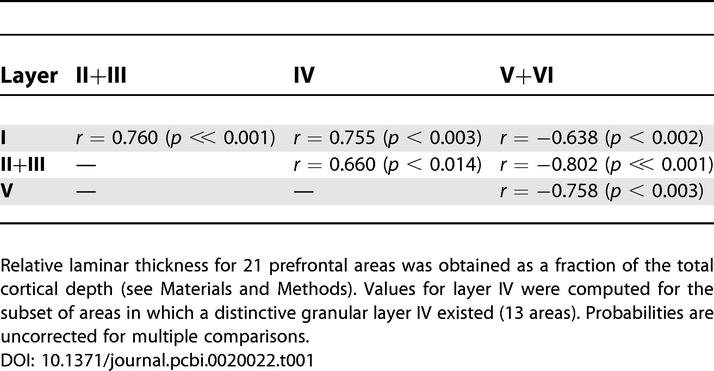
Cross-Correlations of Relative Laminar Cortical Thickness

Correspondingly, the placement of an area in a particular cortical landscape, (sulcal, intermediate, and gyral; see Materials and Methods) had a pronounced effect on the thickness ratio of all upper layers (I+II+III+IV) to deep layers (V+VI). The average ratio for sulcal cortices was 1.8 ± 0.1 (S.E.M.), for intermediate areas 1.3 ± 0.1, and for gyral cortices 1.0 ± 0.1, indicating an almost 2-fold expansion of deep relative to upper cortical layers in gyral cortex compared with sulcal regions.

Interestingly, beyond the differences in relative laminar thickness there were also marked differences in absolute cortical thickness between sulcal and gyral regions (Figure S2). This finding contradicts models which hypothesize that cortical folding only produces isometric changes in laminar thickness. According to the isometric hypothesis, cortical folding affects the ratio of upper to deep laminar thickness, and the shape of neurons (Figure S3), but not the absolute cortical thickness or the absolute number of neurons [10]. Therefore, a simple model for bending neuronal tissue, as outlined so far and depicted in Figure 1C, does not completely explain all experimental observations. We suggest a plausible explanation for this highly regular architectonic feature in the following section.

#### Effect of Folding on Cellular Columns

In view of the marked differences in the relative cortical thickness of superficial and deep layers in gyral and sulcal cortices, we addressed the question of whether they also differed in absolute thickness and the overall number of neurons. We computed the total number of neurons found below 1 mm^2^ of cortex within each laminar compartment using stereological data for neuronal density and the depth of each laminar compartment (for details see Materials and Methods). The isometric folding model [10] presumes that mechanical transformations act on a cortex that has completed its development. Developmental studies, however, indicate that by gestational week 20 in humans, when the primary convolutions are already in place [30], a significant fraction of neuronal migration (about 20%) has yet to occur [31]. The mechanical forces of cortical folding, therefore, may affect cellular passage, particularly of the late developing upper-layer neurons, which must cross the deep cortical layers. We reasoned that tangential compression of the deep layers in gyri would create resistance to cellular passage and may hold back some migrating neurons. In contrast, stretching of the deep layers in sulci might enhance cellular migration to the upper layers [32]. Therefore, in the adult cortex one should find corresponding differences in the distribution of neurons in cortical layers.

**Figure 7:**
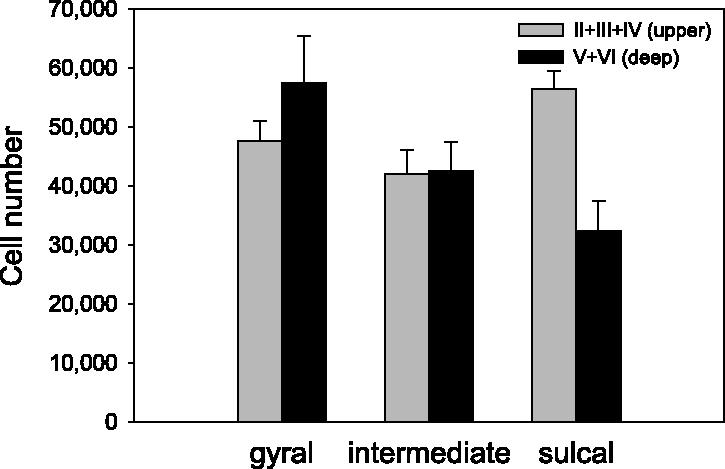
Effect of Cortical Folding on Neuron Number Number of neurons found below a unit of 1 mm^2^ surface area and extending to the variable width of the upper (II+III+IV) and deep (V+VI) layers of gyral, intermediate, and sulcal cortices. Layer I was excluded from the analyses because of its separate developmental path and paucity of neurons [31]. There were significantly fewer neurons in layers V+VI of intermediate areas than in gyral areas and in the deep layers of sulcal than intermediate areas (pairwise *t* tests, Bonferroni-adjusted, *p* < 0.05). Conversely, there were more neurons in the upper layers of sulcal areas than in intermediate or gyral areas.

Our quantitative data support this hypothesis. The average number of neurons found under 1 mm^2^ of cortical surface in different areas was significantly higher in the deep layers of gyral cortices (57,396 ± 7,973) compared to intermediate (42,508 ± 4,823) and sulcal (32,205 ± 5,257) cortices (rank correlation of individual values: ρ = 0.73, *p* < 0.001; one-tailed *t* tests of means gyral > intermediate, intermediate > sulcal, *p* < 0.05, Bonferroni-adjusted for multiple comparisons; Figure 7). This finding was also exemplified by direct comparison of architectonic areas that have distinct sulcal and gyral components, such as areas 13 and 8. The absolute number of neurons in the deep layers of gyral 13 was 52% larger than in sulcal 13, and 64% larger in gyral compared with sulcal subdivsions of area 8.

However, neuronal density in upper and deep cortical layers was not significantly different between gyral, intermediate, or sulcal cortices (analysis of variance, *p* > 0.05). This implies that any addition of neurons to a laminar compartment is associated with a proportional increase of the compartment's thickness. Indeed, there was a significant trend in increasing absolute cortical thickness from sulcal to intermediate to gyral cortices (rank correlation of individual values ρ = 0.56, *p* < 0.01; sulcal average: 1,747 ± 112 μm; intermediate average: 1,791 ± 90 μm; and gyral average: 2,227 ± 132 μm), confirming quantitatively previous qualitative observations [9]. The greater gyral thickness was due to an absolute expansion of the deep layers (sulcal V+VI: 640 ± 70 μm; intermediate V+VI: 788 ± 66 μm; gyral V+VI: 1,140 ± 95 μm; *p* < 0.01), but not of other layers (analysis of variance with post-hoc testing). Therefore, the accumulation of additional neurons in these layers corresponds to a proportional increase in laminar thickness. This finding shows that the absolute number of neurons as well as the absolute thickness of deep gyral layers could be increased as a result of folding during neuronal migration. The aggregation of additional neurons to deep layers also accounted for a significantly higher average number of neurons in cortical columns (across all layers) under a unit surface area in gyral cortices compared with nongyral cortices (*t* test, *p* < 0.03).

Because the orientations of most sulci in the rhesus monkey brain are not orthogonal to the coronal plane, histologic sectioning of the brain cuts some areas at an angle, resulting in a certain degree of error in the measurement of cortical thickness. However, measures for the relative thickness of laminar compartments are unaffected by this confound. Moreover, we verified our results for the absolute cortical thickness with the help of an independent magnetic resonance (MR) image measure, by calculating cortical depth in 3-D, independent of plane of sectioning. This approach was applied to the whole cerebral cortex by examining a computer-reconstructed hemisphere that was based on the average of five rhesus monkey brains (obtained from MR images). Despite noise introduced by the low resolution of thick MR sections (1 mm), this approach also showed a significant correlation between total cortical thickness and cortical elevation (ρ = 0.36, *p* = 0.000001; Figure S2).

### Discussion

Our results provide quantitative evidence consistent with the hypothesis that cortical convolutions are formed by axonal tension exerted across linked cortical areas [5]. Moreover, mechanical forces appear to have a significant role in shaping cortical architecture, supported by the finding that the relative as well as absolute thickness of cortical layers differed between gyri and sulci. The difference in absolute thickness was accompanied by a corresponding difference in the number of neurons in cortical layers, but not their density, suggesting an effect of cortical folding on cell number across the cortical landscape. This finding underscores the fact that cortical columns under a unit surface vary in neuronal number across areas [25], and are not constant as is still widely assumed [33,34]. The systematic trends in the quantitative features of cortical architecture may result from mechanical factors acting at multiple stages during cortical development. We elaborate on each of these points below.

### Axonal Tension and Cortical Convolutions

The quantitative connectional data are consistent with the hypothesis that cortical convolutions are formed by axonal tension exerted between linked areas [5], resulting in predominantly straight projections in the adult cortex. Our results show that the majority of projections in the primate prefrontal cortex had straight trajectories, while only a minority were strongly curved. Moreover, dense connections followed straight paths, and weak connections followed curved trajectories. The average cumulative density of projections originating from any prefrontal area was also dominated by straight or mildly deflected projections, in agreement with the idea of a global competition of axonal tension forces.

Our findings were obtained for the interconnections of prefrontal cortices, and it is not yet known whether cortical projections, in general, follow straight paths. However, long-distance prefrontal projections to parietal or temporal cortices travel in rather narrow corridors in the white matter below the major convolutions, and it is conceivable that they may also take fairly straight or mildly deflected paths. Further data are necessary to address this issue.

The ultimate aim of the axonal tension hypothesis would be to provide an analytical relationship between the organization of connections and the specific shape of the whole cortex. However, this relationship is still unattainable, because information about the exact amount of axonal tension, course of cortical development, spatial layout, and density of projections in the primate cortex is incomplete or, in the case of the human brain, mostly lacking. Consequently, the present data on connections correlate with, but do not provide causal evidence for the formation of convolutions, which may be controlled by other factors. Further data are necessary to determine the specific factors that underlie the generation of convolutions and determine their specific shapes.

In line with the present findings, however, the stability of principal cerebral convolutions may be attributed to comparable cortical connections among individuals within a species. For example, a well-known feature of hemispheric specialization in humans is the more shallow angle between the Sylvian fissure and the base of the brain in the left cortical hemisphere [35,36]. That difference may be related to the tension exerted by the arcuate fasciculus, a massive fiber bundle interconnecting language-related Wernicke's and Broca's areas. If this tract is denser in the left hemisphere [37], in line with its presumed functional importance, it might also be straighter, as were the denser projections within prefrontal cortex. On the other hand, the density of corticocortical projections can vary considerably from one individual to another [38–40], which may help explain the individual variability of tertiary convolutions [41]. Other mechanical processes, such as the buckling of unequally growing, bonded cortical layers [17], may also contribute to tertiary variability.

An alternative interpretation of the data is that convolutions may form first and are only later linked by specific projections. However, there is evidence that experimental lesions disrupting fiber pathways lead to aberrant cortical convolutions, not only locally, but also in remote cortical regions [19,42,43]. By contrast, in other pathological conditions that deafferent cortical regions and alter significantly synaptic function, such as neurodegenerative diseases of adult onset, morphological changes are subtle (reviewed in [44]). The changes in convolutions after lesions, therefore, cannot simply be explained by disruption of synaptic activity following deafferentation. Moreover, synaptogenesis and synaptic elimination are comparatively late developmental events [45,46], and continue to occur at a great rate into postnatal life [47], at a time when convolutions are already in place. In addition, synaptic changes are in constant flux throughout life, underlying learning, memory, and cognitive processes, yet the gross morphology of convolutions does not change throughout the life cycle. Thus, while a variety of pathological conditions and experience alter synaptic activity, axonal transection selectively leads to dramatic changes in convolutions in infants or adults. These observations suggest that cortical projections play an important role in shaping cortical convolutions.

### Folding and Cortical Architecture

Our quantitative findings are also in agreement with the idea that cortical folding has a role in shaping cortical morphology and architecture. In line with the geometrical predictions for an isometric (volume-preserving) folding of the cortical sheet, a correlation analysis showed that the relative thickness of the upper cortical layers was collectively reduced in gyri and increased in sulci. Reverse relations were obtained for the deep layers.

The simple isometric model, however, did not completely explain the quantitative findings of differences in absolute cortical thickness and cell number across the cortical landscape. According to the isometric hypothesis, folding would affect relative laminar thickness, but not the absolute number of neurons within a cortical column; therefore, the thickened deep layers in gyral regions should be less dense. These layers, however, are at least as dense as the deep layers in sulcal cortices [25], resulting in a significantly higher average number of neurons in cortical columns in gyral regions than in sulcal regions. These findings, therefore, challenge a simple isometric transformation, as well as the frequently assumed constancy in the number of neurons of a cortical column under a unit surface of cortex across different areas [33,34].

What mechanism underlies the increased number of neurons in deep gyral layers? We suggest that the increase reflects a dynamic interplay of developmental events and mechanical forces. As the cortex folds, tangential forces cause vertical expansion of the deep gyral layers, increasing their volume, and allowing additional aggregation of neurons. This interpretation is consistent with a near doubling of neurons in the expanded deep layers in gyri in comparison with sulci. Conversely, migration to the upper layers may be facilitated in sulcal regions, a prediction supported by our data (Figure 7). Another implication of this hypothesis is that greater cortical curvature should result in larger forces and consequently in an increased accumulation of neurons and greater cortical thickness. This idea appears to be supported by a significant rank correlation between gyral curvature and cortical thickness in human brains [48].

Traditionally, cortical areas have been distinguished by their individual and characteristic cellular architecture (e.g., [25,49,50]). We showed that, in addition, the architecture of cortical areas is systematically affected by their location within the convoluted cortical landscape. Moreover, when a cortical area has distinct gyral and sulcal components (such as areas 13 and 8), the number of neurons in the component parts varies according to the pattern seen for other gyri and sulci. This evidence suggests that a genetic model for specification of architectonic areas alone cannot fully explain the differences in cell number in sulci and gyri in the same architectonic area. The architecture of cortical areas, therefore, represents a combination of features resulting from individual development as well as mechanical shaping processes.

### Comparison of Cortical Folding Models

Many attempts have been made to understand the factors underlying the formation of cortical convolutions [5–7,9,11,15–19,21,51,52]. We consider briefly three representative models. The model of laminar buckling [17] proposes that cortical convolutions arise from the unequal growth and surface size of bonded cortical layers. This model accounts quantitatively for the general size and frequency of convolutions in normal human brains and in malformations of lissencephaly and microgyria. However, the model does not explain why the placement of the major convolutions is comparable among normal individuals, rather than random. Moreover, the model does not account for the differences in total cortical thickness in the cortical landscape.

Another hypothesis suggests that convolutions are formed through active growth [7], as specified by genes (e.g., [21,53,54]). As a contributing factor, unequal regional growth would result in cortical buckling, through a buildup of intrinsic tension in the cortical sheet [11]. Gyri would form in regions of greater thickness and sulci would arise as the “faultlines” along thinner regions, thus reproducing the observed differences between thicker gyral and thinner sulcal regions.

Neither laminar buckling nor active growth can explain why convolutions are modified after destruction of connections [19,43]. This observation is explained by the axonal tension model of cortical folding [5], which suggests that the characteristic landscape of convolutions is produced by the nonrandom organization of cortical connection networks [55]. The model accounts for the trajectories of cortical projections analyzed here, and the observation that there are significantly more connections within than between gyri [56]. The basic version of this model [5] assumes an initial cortical sheet of uniform thickness that undergoes isometric transformation as it folds. As discussed above, such a mechanism may explain systematic differences in relative laminar thickness, but cannot explain the differences in absolute cortical thickness between gyri and sulci. The consistent and significant differences in absolute cortical thickness and cell number described here could arise if cortical folding partly overlaps with neuronal migration during late gestation, through active growth, or selective cell death. We further explore these possibilities in view of developmental events below.

### Relationship of Cortical Architecture and Connections to Developmental Events

#### Cell death and axonal pruning.

One possible explanation for the inverse relationship in cell number in the upper and lower layers of gyri and sulci is selective cell death during development. In that scenario, cell death would have to be biased for neurons in deep layers of sulcal cortices and neurons in superficial layers of gyral cortices. While there is no evidence that cell death mechanisms act in this specific manner in the cortex, we can entertain the intriguing possibility that lateral stretching of neurons (in superficial gyral and deep sulcal areas), but not lateral compression (in deep gyral and superficial sulcal areas) may be a contributing factor to cell death (see Figure S3). Both possibilities can be tested in future developmental studies. Data from a variety of species, however, indicate that cell death is an early regressive event in development (reviewed in [45,46]). This evidence suggests that cell death may not contribute to sculpting the supragranular cortical layers, in particular, which develop late (for reviews see [57,58]).

Another key regressive event in development is the elimination of exuberant axons [59], which likely contributes to establishment and modification of convolutions as well. Convolutions are thus formed in development when proliferative and regressive events are taking place, so the conditions differ from the adult state. Nevertheless, there is evidence that the predominance of straight connections is also present in development [60] as seen here in adult monkeys.

#### Neuronal migration, connections, and cortical architecture.

The mechanical model offered here to explain differences in cell number in gyri and sulci relies on a partial overlap of neuronal migration, formation of connections, and cortical folding. In this context, data available for macaque monkeys show that corticocortical and callosal connections are formed prenatally [60–64], as are cortical convolutions [65]. The timing for the establishment of connections varies from early midgestation in limbic areas [62] to late development of callosal projections [66], indicating a highly variable timing of axogenesis across cortical areas that partly overlaps with cortical migration [67]. In macaque prefrontal cortex, for example, callosal neurons extend an axon while still in the process of migrating [66].

The above observations are consistent with a scenario in which migrating neurons form axonal connections as they arrive in their target layers, contributing to cortical folding through axonal tension. The folding processes, in turn, may influence subsequent neuronal migration, particularly of late-migrating neurons, and result in the systematic differences in the number of neurons in upper and lower layers of sulci and gyri, as observed here. Additional developmental studies are necessary to resolve this intriguing finding and to further evaluate distinct developmental models.

### Genetic Specification and Mechanical Self-Organization

The idea that the morphology of the brain is shaped through a dynamic interplay of genetic factors and mechanisms of physical self-organization has a long tradition in biology. In the late 19th century, for example, Wilhelm His “modeled” the morphological development of the brain by deforming rubber tubes and clay plates and explained the cerebral shape by mechanical buckling resulting from unequal growth and competing volume demands of different brain structures [6,11]. The work of His and other embryologists gave rise to the concept of Entwicklungsmechanik (developmental mechanics), which emphasized a causal sequence of developmental events steered by physical forces. Physicochemical concepts of pattern formation, which build on the classic foundations laid by His [6], D'Arcy Thompson [8], and Henderson [68], are now valued and applied in many areas of biology (e.g., [69–71]). However, such concepts have been largely ignored in neuroscience during the last decades in favor of molecular and genetic approaches. The recognition of physical factors in brain morphology, however, offers an exciting opportunity for new computational approaches of modeling brain development based on physical principles.

A feasible scenario for integrating genetic and physical perspectives of cortical development is that genetic factors underlie the timing of development of different cortical layers and areas, and mechanical factors come into play as the cortex grows and is linked, resulting in the self-organization of migrating neurons, cortical layers, and cortical convolutions. Thus, a dynamic interplay of genetics and simple physical principles discussed in various mechanical models may underlie the formation of cortical convolutions as well as cortical architecture. Such a scenario may explain why primary and secondary convolutions are highly characteristic and placed consistently within a species [6,17], but tertiary convolutions are more variable [7,41].

Our results suggest that systematic differences in cortical morphology among individual brains [53,54] might also offer clues on the variability of the underlying connectivity. This relationship should be explored particularly for cortical malformations, such as in microgyria [72], or when developmental structural impairments are suspected, for instance, in schizophrenia [48], autism [73], and Turner syndrome [74].

## Materials and Methods

### Animals and prefrontal injection and projection sites.

Analyses were based on quantitative neuroanatomic data systematically detailing the densities and trajectories of corticocortical projections in prefrontal cortices of adult rhesus monkeys *(M. mulatta).* Experiments involving animals were conducted according to the NIH guide for the Care and Use of Laboratory animals (NIH pub. 86–23, revised 1996), and experimental protocols were approved by the Institutional Animal Care and Use Committee at Boston University School of Medicine, Harvard Medical School, and New England Primate Research Center.

Data were compiled from tract-tracing experiments (23 injections of retrogradely transported tracers, HRP-WGA, or distinct fluorescent dyes, in prefrontal cortices of 21 animals). Injection sites included 11 of 22 prefrontal areas and their subdivisions that we previously identified in rhesus monkeys [75], including lateral (*n* = 11; dorsal and ventral area 46, dorsal area 8, and lateral area 12), orbital (*n* = 9; orbital area 12, area 11, area 13, area OPro, and area OPAll/OPro) and medial (*n* = 3; area 32 and medial area 9) areas. Detailed analyses of the cortical connections of these areas were described previously for studies unrelated to the present study [22–24]. Data were obtained from exhaustive mapping of systematically sampled coronal sections (one in every 20 sections) throughout the prefrontal cortex. The database included all afferent projection sites and the number of projection neurons within each projection site. Projection neurons were found in all prefrontal architectonic areas and their subdivisions (cumulative *n* = 289 sites; 123,866 projection neurons).

### Retrograde tracing and normalization of neuronal density.

The retrograde tracing method is ideal for addressing the proposed issues, since each projection neuron is labeled by transport of tracer through its axon from the cortical injection site back to the parent cell body. The incidence of a neuron projecting to two areas is low in the prefrontal cortex [76,77]. A count of labeled neurons, therefore, provides a good estimate of projection density. Anterograde tracing, on the other hand, labels branched terminations of axons and is not suitable for estimating the number of axons involved in each projection.

The absolute number of labeled neurons varies, depending on size of injection, tracer type, etc. In order to account for such variability, absolute projection densities were normalized within each case to yield the relative density for each projection site (e.g., total number of neurons in a given case: 2,000; labeled neurons at site x: 200; relative density of projection x = 10%.). We refer to this proportion as (relative) projection density.

### Classification of projection sites into straight, intermediate, and curved.

Maps obtained from coronal brain sections (40 or 50 μm thick) were used to classify all prefrontal projection sites into three trajectory classes, according to the following criteria. If *x* was the length of the shortest path between projection origin and termination, and *d* the maximum deviation of the actual trajectory, measured perpendicular to the shortest path, the curvature index, *c,* with *c* = *d*/*x,* defined the trajectory categories as follows: straight for projections following the shortest path (*c*_st_ < 0.1); intermediate for projections slightly deflected by an intruding sulcus (0.1 ≤ *c*_im_ < 0.33); and curved if the projections were completely bent around a sulcus (*c*_cu_ ≥ 0.33; Figure 2).

The classification of projection sites into each of the above three categories was based on the shortest possible path in 3-D linking each projection site to the injection site. Direct visualization of axonal trajectory is possible close to the injection site, where labeled axons emerge as a bundle, and for areas where the origin and destination are in close proximity (e.g., Figures 3 and 4). Labeled axons traveling a long distance initially emerge from the injection site in a bundle and then take multiple trajectories en route to their respective sites of origin, which exceeded 15 areas for some cases. The intermingling of axons traveling in multiple directions and the distribution of projection origins and terminations over multiple sections do not allow a metric mapping of the curvature for all individual axonal trajectories with confidence. Therefore, we classified each projection site into one of three trajectory classes based on the shortest likely path, which was confirmed by direct observation, though this was not often possible.

The deviation of projections from a straight line was determined by consideration of all “obstacles” between injection site and projection site, including sulci, dimples, and the underlying striatum (caudate and putamen). Corticocortical axons are known to travel around the striatum, but cortico-subcortical projections cut through the striatum, forming the internal capsule, which bisects the striatum into the caudate medially and the putamen laterally (Figure 4C). The curvature of the trajectory of axons from origin to termination was estimated as the maximal deviation from a straight line along the entire axonal trajectory, spanning all mapped coronal sections between each set of points. In some cases a cluster of projection neurons had a straight trajectory and another cluster of neurons in another part of the same architectonic area had a mildly deflected trajectory. In such cases the entire projection site was considered to be intermediate. An example of the latter case is shown in Figure 4C and 4D.

### Relationship of laminar morphology to convolutions: Quantitative prefrontal architecture.

We used unbiased stereologic procedures to estimate normative structural data, including laminar thickness and density of neurons by layer. Normative data were obtained from several coronal sections through the prefrontal cortex in each of seven brains to estimate neuronal density. The sample size exceeded by more than three times [25] the sample size required by approaches of unbiased stereologic counting [78]. The borders of architectonic areas were delineated on the basis of a parcellation scheme [75] modified from the map of Walker [79].

### Absolute and relative laminar thickness.

To determine to what extent cortical folding affects cortical architecture, it was necessary to determine cortical thickness and neuronal density by layer for all 21 prefrontal architectonic areas (the only area not considered was dorsal area 10, which is very similar in architecture to medial area 10 that was included in the sample). The absolute thickness of cortical layers (compartmentalized into layers I, II+III, IV, and V+VI) in 21 prefrontal cortices was measured from coronal sections. Thickness measurements of laminar compartments were obtained through the central portions of sulci or gyri, avoiding the extremes of the convolutions, such as the crease of sulci, or the maximum convexity of gyri, where the effect of folding on laminar thickness is greatest (Figure 6). Because the orientations of most sulci in the rhesus monkey brain are not orthogonal to the coronal, sagittal, or horizontal planes, slicing of the brain is likely to cut some areas at an angle, resulting in a certain degree of error in the measurement of cortical thickness. However, the ratio of the upper layers to the deep layers (expressing relative cortical thickness) is constant regardless of random or systematic error of absolute cortical thickness. We calculated the relative thickness of each laminar compartment by normalizing its absolute thickness by the total cortical thickness.

### Neuronal density.

We used unbiased sampling procedures to estimate neuronal density, which is based on the principle that each neuron has an equal opportunity of being counted. Using this method, each architectonic area was outlined and subdivided into laminar compartments. Density of neurons within each laminar compartment for each area was measured from sampling windows of 50 × 50 μm taken randomly according to stereological procedures, as described previously [25]. Neuronal density data were obtained outside the extreme points of the cortical landscape, such as crests of gyri and depths of sulci. Values from the randomly sampled windows were used to compute the density of neurons for each laminar compartment in each area, calculated as neurons/mm^3^.

### Overall number of neurons in laminar compartments or entire cortical columns.

We computed the absolute number of neurons found below 1 mm^2^ of cortical surface extending to the entire (and variable) width of the laminar compartment, by combining data on neuronal density (as described above) and laminar thickness within each laminar compartment of each prefrontal area. The information about the absolute number of neurons under 1 mm^2^ of a particular area, therefore, represents an average value for the whole area, unaffected by local variations in curvature. The latter values made it possible to compare the number of neurons found in laminar compartments in sulci and gyri.

### Classification of prefrontal cortical areas into gyral, intermediate, and sulcal.

To investigate the relationship of laminar thickness to cortical convolutions, we classified prefrontal areas as predominantly gyral, sulcal, or intermediate (Table 2). The intermediate category comprised cortical areas of mixed character, or areas that had straight parts as well as a shallow sulcal component. Note that this categorization is independent from the classification of trajectories into straight, intermediate, and curved classes.

**Table 2:**
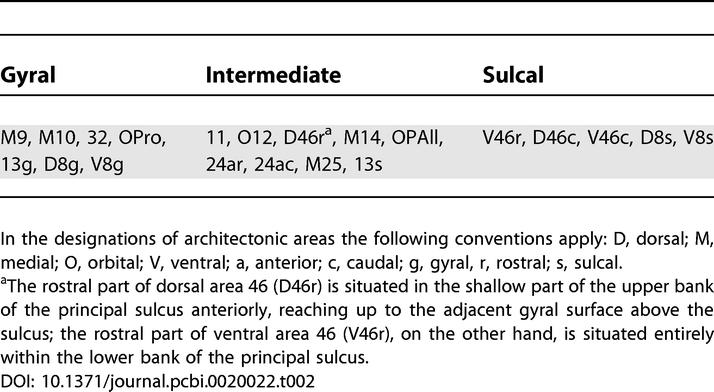
Classification of Prefrontal Areas Based on their Curvature

### Bending a slab of layered neural tissue.

The problem of bending the 3-D cortical sheet can be simplified by considering a 2-D slab of tissue which consists of two layers and is bent into an annulus (Figure 1C). For simplicity, the two laminar compartments are assumed to have the same thickness and identical material properties, and the slab, which contains densely packed cells and fibers, can be considered incompressible. Thus, the area of the slab remains the same after bending, and this is also true for both layers individually:


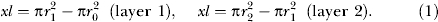


The thickness of each layer after bending is described by:


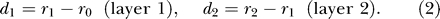


By factorizing the right-hand sides in Equation 1 and substituting *d*_1_ and *d*_2_ (the laminar thicknesses after bending) from Equation 2 one derives:


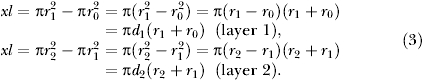


Using the two new expressions for the original area, which is identical for the two layers, one derives the ratio of the thicknesses *d*_1_ and *d*_2_ after bending:


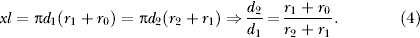


From the construction of the annulus it is known that


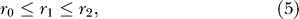


because the bending was effected with layer 1 towards the center and layer 2 on the exterior. Finally, from Equations 4 and 5 follows that


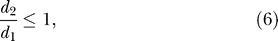


that is, layer 2 becomes thinner after bending, relative to layer 1. The reverse would be true if the annulus was formed by bending the slab with layer 1 on the outside.

## Supporting Information

Figure S1 Three Views of the Anterior Half of the Rhesus Monkey Brain Showing the Architectonic Areas of the Prefrontal Cortex (A) Medial areas; (B), lateral areas; (C) orbital areas. Numbers show architectonic areas. A, arcuate sulcus; Cg, cingulate sulcus; OLF, olfactory area; P, principal sulcus; PAll, periallocortex; and Pro, proisocortex. The map is based on the parcellation of Barbas and Pandya [75].
(361 KB TIFF)

Figure S2 Cortical Thickness Depends on Cortical Elevation Original (A) and inflated (B) thickness maps with color scale indicating cortical thickness (red, thickest; green, thinnest). While sulcal regions are buried in the folds in (A), they are revealed in (B). By comparing the two images, is it apparent that thinner cortical regions correspond to sulci. T1-weighted 3-D spoiled gradient–recalled image (SPGR; TR70, TE6, Flip 45).
We investigated the global relationship between the total thickness of cortical areas and cortical elevation. MR images of the brain were obtained from five rhesus monkeys. MR was performed with a 1.5T superconducting magnet (Signa; General Electric, Milwaukee, Wisconsin, United States) at Brigham and Women's Hospital, Boston, Massachussetts, United States. The monkeys were sedated with a mixture of ketamine (10 mg/kg) and rompun (1.25 mg/kg), and positioned in a nonmetallic stereotaxic device. A T1-weighted 3-D spoiled gradient–recalled image (TR70, TE6, Flip 45) was obtained through the brain, using a 512 × 384 matrix and a 16 × 16 field of view (scanner voxel: x_size = 0.46875; y_size = 0.46875; z_size = 1).
The Freesurfer software package (http://surfer.nmr.mgh.harvard.edu) was used to reconstruct each individual cortical map in 3-D [80,81]. We then used the five reconstructed brains to generate an average template hemisphere, a probabilistic topographical map and to compute the average cortical thickness ([82]; Figure S2A). As part of the reconstruction routine, the cortical surface was represented as a smoothly deformed ellipsoid, allowing clear view of the cortex buried in sulci (Figure S2B). During the ellipsoid deformation a transform variable kept track of the shift of all surface vertices and recorded negative shifts for gyral points and positive shifts for sulcal vertices to create a topological map of the surface. Despite various sources of variability, the transform variable could be considered an indicator of the gyral or sulcal character of a surface point. In addition, an approximated measure of cortical thickness was computed for each point on the surface [83].
We rank-correlated the gyral/sulcal transform variable with the thickness of all surface points of the reconstructed hemisphere and found a significant correlation between the gyral or sulcal character of a vertex and the absolute thickness of the underlying cortex (ρ = 0.36, *p* = 0.000001). The low correlation may have resulted from the fact that gyral crests and sulcal creases represent only the most extreme cases of cortical folding, whereas large cortical regions, even those forming the walls of sulci or the plateaus of gyri, are relatively flat (compare with Figure 6). Under these circumstances, it is noteworthy that the quantitative relationships for normative structural data described in the main text were obtained, even though extreme regions of cortical folding (such as the crease of sulci and the crest of gyri) were excluded from the unbiased anatomical measurements of laminar thickness and cellular density.
(3.3 MB TIFF)

Figure S3 Folding Shapes Cellular Morphology (A) Low-magnification overview of coronal section through the primary motor cortex, M1. Tissue was processed for SMI-32, an antibody to an intermediate neurofilament protein that labels largely pyramidal neurons in some cortical layers (most densely in lower part of layer III and layer V, and to a lesser extent in layer VI). The large pyramidal neurons in layer V are Betz cells, characteristic of primary motor cortex, seen at high magnification in (B), (C), and (F). Note the differences in the shape of the Betz cells along the convolutions.
(B) High-magnification view of layer V at the crest of a gyrus in M1.
(C) High-magnification view of layer V in a straight gyral part of M1.
(D) Low-magnification photomicrograph of coronal section through the sulcal part of M1 stained for Nissl. Note the sharp edge of layer VI with the white matter in the depths of the sulcus, and the squeezing of the deep layers.
(E) Low-magnification of coronal section matched to the section in (D) processed for SMI-32.
(F) High-magnification view of the deep layers in a sulcal stretch of M1 showing labeled neurons in layer V, and a flat-shaped pyramidal neuron in layer VI. Roman numerals for layers in (A), (D), and (E) are placed at the beginning of each layer that can be distinguished with each stain. Axes: medial is to the left; dorsal, on top.
As suspected previously [5,10], the mechanical impact of cortical folding appears to extend to the finer morphologic features of the cerebral cortex, affecting cellular and dendritic shape in different cortical layers. Morphological modifications of layers, somata, and arbors are also likely of functional significance [84–86] and may result in different modes of functioning in gyral and sulcal regions [7]. The structural influences on cellular morphology and their relationship to function need to be explored quantitatively at the experimental level as well as by modeling approaches. Figure S3 presents an example to demonstrate the differential effect of folding on neuronal arbors residing in upper and deep cortical layers of the same primate cortical area, primary motor cortex, M1.
(5.4 MB PDF)

## Abbreviations

HRP-WGA: horseradish peroxidase–wheat germ agglutinin
MR: magnetic resonance
OPAll: orbital periallocortex
OPro: orbital proisocortex
