# Supplementary material for: Role of Mechanical Factors in the Morphology of the Primate Cerebral Cortex
Source: PLoS Comput Biol. 2006 Mar 24;2(3):e22. doi: 10.1371/journal.pcbi.0020022 (PMC1409812; doi:10.1371/journal.pcbi.0020022)
Supplement: Supplementary file 1 [file pcbi.0020022.sg003.pdf]

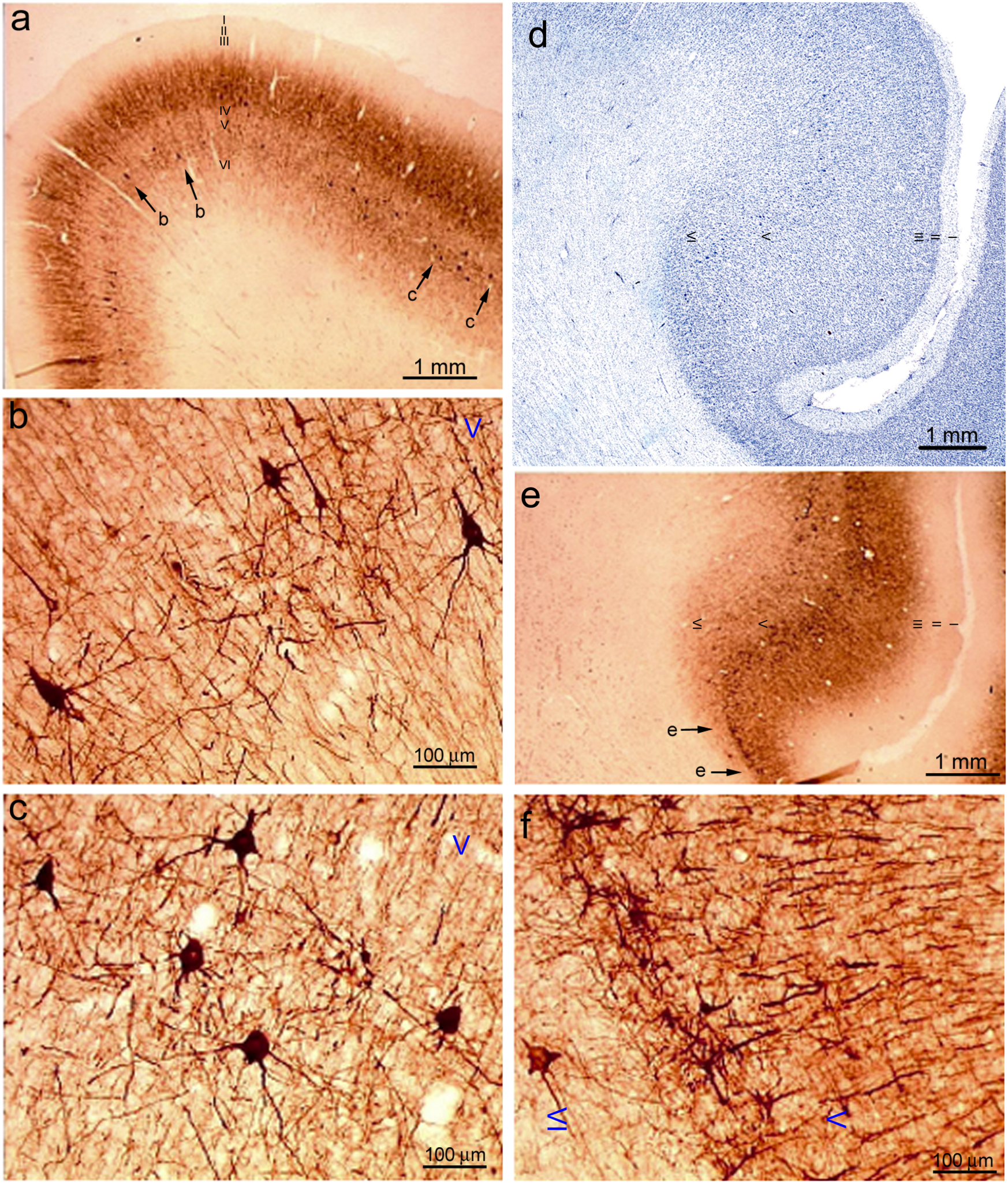
a
I
II
III
IV
V
VI
b
b
c
c
1 mm
b
V
100 µm
c
V
100 µm
d
1 mm
e
e
e
1 mm
f
100 µm
